# A leadership ethics curriculum: Bringing mixed-methods interdisciplinary insights to the ethical complexities of health leadership

**DOI:** 10.1177/08404704251329480

**Published:** 2025-04-09

**Authors:** Schuyler Pringle, Randi Zlotnik Shaul, Ema Rosa, Bonnie Au, Lennox Huang

**Affiliations:** 1 7938 University of Toronto, Toronto, Ontario, Canada.; 2 7979 The Hospital for Sick Children, Toronto, Ontario, Canada.

## Abstract

In response to the increasingly complex ethical issues facing health leaders, the Bioethics Department at The Hospital for Sick Children (a Canadian quaternary care paediatric research institution) was asked by senior leadership to develop a leadership ethics curriculum that would further develop the ability of its institution’s leaders to deliberate and make morally defensible decisions in their roles. Insights from an interdisciplinary literature review suggest that the general objectives and structure of leadership ethics teaching remain constant, with specifics changing depending on the organization and intended participants. Implementing findings from an institutional needs assessment, our modular leadership ethics curriculum, which engages participants in asynchronous and synchronous learning, was designed to support (1) understanding of personal and organizational values, (2) recognizing the significance of attending to the ethical dimensions of decisions, (3) familiarity with leadership and organizational expectations, and (4) practicing application of ethical analysis, enhancing abilities and confidence to engage with ethical issues.

## Introduction

Leadership in contemporary healthcare can require making decisions that result in both *moral distress* and *stress* for those one leads and for leaders themselves.^1-5^ Ethical leadership is essential for responding to time-sensitive, critical scenarios,^2,5^ as well as for the day-to-day delivery of quality healthcare.^1,2^ In March 2023, the Bioethics Department at The Hospital for Sick Children was asked by its senior leadership to create a leadership ethics curriculum that would enhance the abilities of institutional leaders to deliberate and make morally defensible decisions about the ethically complex issues they face in their healthcare roles. The many dimensions of ethical leadership were also explored by the hospital’s Bioethics Advisory Committee. Members underscored that the development of a high-quality leadership ethics curriculum should be prioritized. Accordingly, a team with expertise in bioethics, leadership education, and research methodology designed a leadership ethics curriculum to support our hospital leaders. Our author team reflects the roles that contributed to the curriculum’s design—from recognizing the need for the course, to data gathering and analysis.

**Figure 1.**
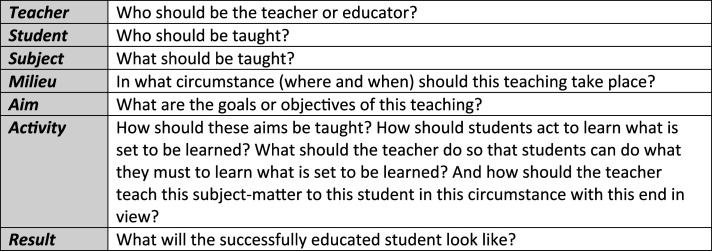
J. T. Dillon’s seven questions that educators must answer in order to prepare a course curriculum.

Education scholar J. T. Dillon argues that there are seven questions that educators must answer in order to prepare a course curriculum (see Figure 1).^6^ Given the original request from senior leadership, our curriculum development team began its design process with partial answers to four of the seven questions. The *students* (i.e., “participants”) of the course are the formal and informal leaders within our institution. The course’s *subject* is “leadership ethics,” with the principal *aim* of facilitating morally defensible decision-making by participants in the value-laden situations that healthcare institutions have faced in recent years. And, finally, the successful participant is better equipped, and more confident, in their ability to make ethically defensible decisions. Beyond this, Dillon’s seven questions remained unanswered, requiring further research.

This article explains the steps we took to answer Dillon’s seven questions and, thereby, develop our hospital’s leadership ethics curriculum. This dissemination is significant because rather than present a single institution’s course contents, we aim to present a transferable model curriculum that may be tailored to distinct cohorts and the unique contexts of other institutions.

## Methodology

Our approach to curriculum development consisted of four stages: (1) reviewing of the contents and structures of contemporary North American leadership ethics training programs; (2) reviewing of literature on leadership ethics education; (3) conducting an institutional leadership needs assessment about leadership education; and (4) creating of a *versatile* curriculum—adaptable to evolving priorities and varying contexts. This section summarizes the methods used in stages 1-3.

### Stages 1 and 2: Comparative analysis and literature review

Our team began by collating the publicly available features of contemporary North American leadership ethics training programs. To be included in the review, the course needed to be *currently* offered, as a *standalone* program on *leadership ethics*, with information about aims, contents and/or structures *publicly available* on a web-based platform. Courses were accessed through on-line search engines. Search terms included variations of “leadership ethics training program” and “leadership ethics course.” The intent was to learn from the programs currently advertised publicly. However, because public access to such information was limited, our curriculum development team ultimately focused on the explicitly expressed learning goals and general themes of advertised modules. This course information was then analyzed using an inductive approach, allowing themes and topics to emerge from the data. Initial patterns were subsequently identified, and topics iteratively developed, refined, and/or added to capture emerging insights.

Findings from the comparative analysis (see Figure 3) were then compared with formal literature on leadership ethics education in business and healthcare. While the foundational values guiding healthcare decisions are distinct from those within business,^7,8^ *the pedagogy* used in leadership ethics education in both business and healthcare have much in common. Leadership ethics teaching in business aims to enhance the ability of participants to make morally defensible decisions—the principle aim of our curriculum. To clarify this pedagogical approach of leadership ethics, we turned to the work of Ronald R. Sims (recognized as one of the most influential authors on ethics pedagogy in business^10^), for not only is Sims uniquely focused on the actual *design* of a leadership ethics curriculum, but there is also demonstratable overlap between Sims’ pedagogical teachings and the leadership ethics pedagogy already employed in healthcare.

### Stage 3: Leadership needs assessment

**Figure 2.**
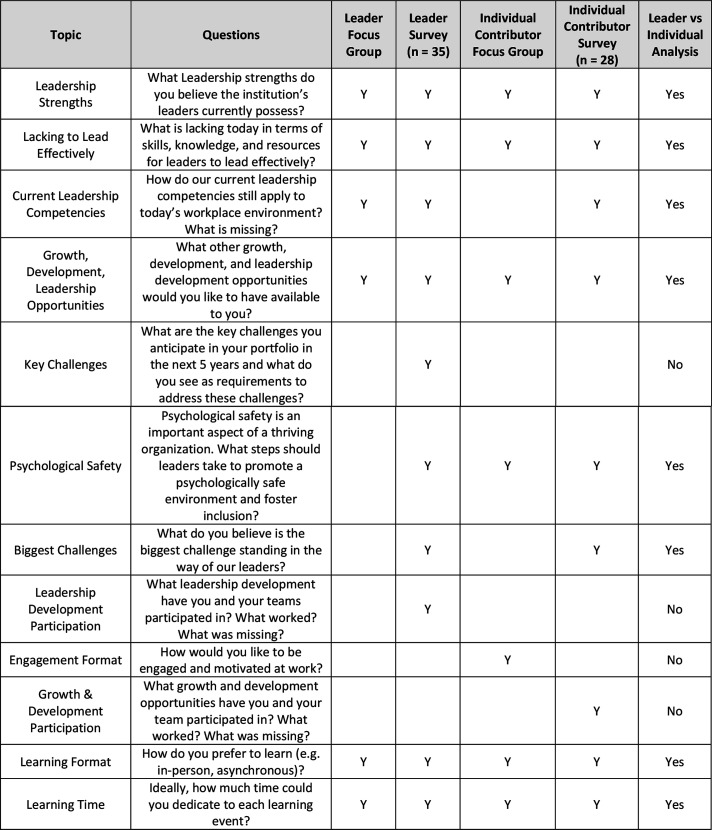
List of leadership needs assessment questions.

An invitation to participate in a leadership needs assessment was sent to all individuals in our institution holding a *formal* leadership role. The needs assessment was facilitated by organizational development professionals and senior hospital leaders. While the needs assessment was designed with a broad focus, we analyzed the data generated to capture themes relevant to ethics. The following questions (Figure 2) were asked of leader-participants to determine the future needs of leaders to lead effectively within our institution. Subsequent in-person focus groups and surveys were conducted with individuals not occupying a formal leadership role in order to acquire their perspectives on the leadership behaviours required for leaders to lead effectively.

Responses were analyzed using an inductive approach, allowing themes and topics to emerge from the data. Initial patterns were identified, and topics were iteratively developed, refined, and/or added to capture emerging insights. To quantify the findings, the frequency of each theme or topic was calculated and divided by the total number of responses, allowing us to see the prevalence of topics in the data.

## Findings

The findings of the needs assessment reinforced the original request by senior leadership to develop a leadership ethics curriculum. Few participants responded that “ethics” or “ethical decision-making” are strengths currently possessed by our hospital leadership—with many indicating that such skills are lacking; accordingly, an overwhelming majority expressed that formal leadership ethics training is highly needed. This section summarizes the findings from the literature review and needs assessment in relation to Dillon’s seven curriculum questions (Figure 1).

### Question: Aim

The literature review suggests that all organizational ethics teaching efforts ought to include the following three learning goals. First, a leadership ethics course ought to broaden its participants’ understandings of ethics and its complexities, and heighten their awarenesses of the ethical accountabilities they owe in being a leader at their organization.^11^ Ethical decision-making can be a complex process—one must weigh the often conflicting values of invested parties, sometimes resulting in a “lose-lose” situation, with negative repercussions.^11^ To be an effective leader, one must be able to embrace such situational ambiguity.^5^ Given value pluralism, leadership ethics teaching within business focuses on providing participants with a theoretically and experientially sound conceptual framework for *evaluating* the various ethical aspects of a situation according to personal and institutional values, and then for *making their* decision.^11-13^ Accordingly, the good decision does not only effectively address the problem, but is based on relevant, well-justified values.^14^ As required by Accreditation Canada of Canadian hospitals, our institution has an ethics framework, which is employed in the leadership ethics curriculum tailored to our institution’s context. Such institution-wide frameworks are generally informed by a range of ethics frameworks in the healthcare literature.^14^

However, most decisions cannot be subject to such rigorous analysis. The Dual Process Theory (DPT) of human thought and decision-making is used widely, within fields of psychology, sociology, and medicine.^15-18^ Generally, DPT asserts that human thought consists of system 1 “fast thinking” and system 2 “slow thinking.” Individuals who work in a fast-paced, high stress environment (such as healthcare) must rely on fast thinking and, thus, are at a heightened risk of perpetrating the biases or uncritical assumptions influenced by their peers, parents, and/or cultural, personal, and religious backgrounds.^13,17,19,20^ Accordingly, the second and most important goal of any leadership ethics program is to assist participants in understanding the values that genuinely guide their “fast” decision-making—to challenge these values and, thereby, assist participants in feeling confident that the values that they will implicitly rely on going forward are the product of considered reflection, an awareness of the relevant ethical discourse of the day, and an appreciation of the trade-offs associated with different options and approaches.^11-13,19,20^

Finally, participants should appreciate that completing their course in leadership ethics does not in and of itself make them an ethical leader.^11^ The third goal is that an effective leadership ethics course curriculum helps participants appreciate that an ethically accountable leader continuously gives attention to the development of their ethical sensitivities and accountabilities, and that what is considered ethical is closely tied to evolving contexts.^11,20^ Thus, a leadership ethics curriculum requires participants to become comfortable engaging with ethical issues and existing “hidden curriculums”^21^ familiar to their organizational contexts, and to have access to the resources wherein they can continue to discuss ethical issues.^11^ Accordingly, participants need to learn to share their moral understandings and to familiarize themselves with their institution’s ongoing ethics resources.^11,12,22^

**Figure 3.**
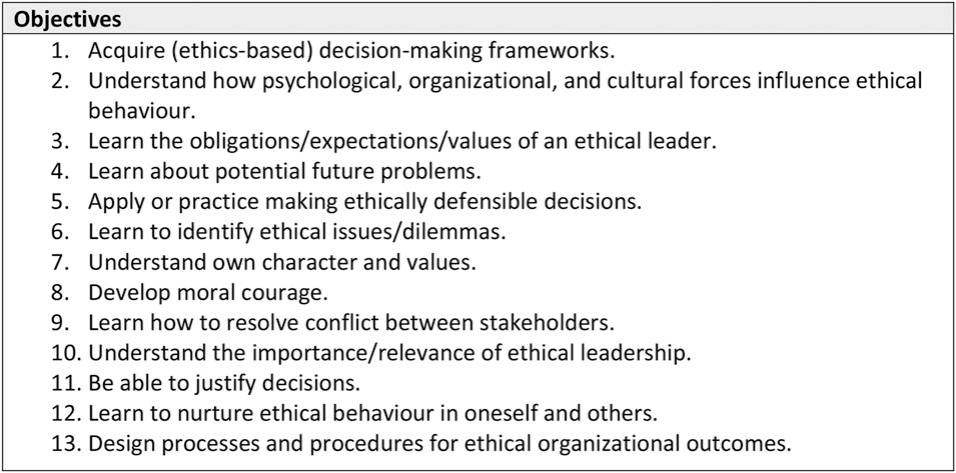
The findings of the comparative assessment: the common learning objectives of contemporary North American leadership ethics training programs.

These broad goals of leadership ethics teaching in business are consistent with those found in our comparative analysis (see Figure 3). In their own review of forty articles (published between 2010 and 2021) on ethics education for healthcare professionals, Henrik Andersson et al. endorse similar objectives.^9^ What is unique, then, about distinct offerings, is how the curriculum is tailored to its learners’ articulated needs and specific contexts. On this matter of ensuring relevance, Sims proved to be particularly insightful.

### Question: Subject

To ensure relevance, Sims stresses that curriculum design should come *after* an anonymous survey of interest-holder views about specific course-objectives and course-design alternatives, followed by power-free focus group discussions.^12^ Such open and active participation of the interest-holders is necessary not only to avoid obstacles associated with participant working situations and mindsets, but also obstacles associated with faculty and competing institutional offerings.^12^ It was on this basis that our design team utilized the leadership needs assessment. From its data, we generated a list of ethical issues relevant to potential participants (including staff burnout and “quiet quitting,”^23^ the impact of one’s personal values-based decisions on the institution’s reputation, ransomware attacks, hybrid work expectations, and compensation) to be used as topics for course discussion.

### Question: Milieu

The leadership needs assessment also found that most leader respondents (37%) preferred to learn in-person, and that most (43%) could ideally dedicate 1 hour to each leadership ethics learning event. That said, 57% indicated that they preferred to learn *not-strictly-in-person* (i.e., by either a hybrid or a virtual means), and 57% indicated that they could ideally dedicate 2 or more hours to learning events. Leaders also expressed it beneficial to share their experiences with persons that represented a variety of roles, from across the institution; and those opportunities to practice working through ethical issues with a variety of colleagues is lacking within the institution.

### Question: Activity

Given that moral judgement is *action* oriented, and that the primary goal of learning ethical leadership (from the participant’s perspective) is to become more skilful and confident in addressing the kinds of organizational situations that they might encounter, Sims argues that participants ought to engage in *experiential learning exercises*—games, simulations, role plays, or live case studies.^24^ The pedagogical benefits of experiential learning is widely recognized and used to teach ethics both within healthcare,^25-29^ and beyond it.^30-33^ However, regardless of the experiential exercise employed, Sims argues that learning needs to conclude with a debriefing session.^24^ The debriefing phase “is [that] part of the [learning] process in which the reflection takes place and from which the change in the person will occur.”^34^ According to Sims, debriefing can be understood as a dialogue between the instructor/debriefer and the participants; he stresses that it is important to establish a structure that provides a framework to contain the discussion.^24^ We found that something like *Debriefing for Meaningful Learning* is useful.^35,36^ Both the experiential learning exercise and the debriefing phase requires a psychologically safe learning environment.^24^ Sims argues that the facilitator is key to creating such an environment.^24^

### Question: Result

Finally, a leadership ethics curriculum requires an evaluation framework that produces reliable data that can be used to continuously improve upon the course’s efficiency and effectiveness.^24^ However, success should not be measured by the creation of an ethical leader: Ethics education cannot itself turn the immoral individual moral,^37^ for ethical behaviour requires other elements, such an ethical organizational culture.^38^ Therefore, success should be determined by participants being better equipped, and feeling more confident in, their abilities to engage and make ethically defensible decisions.

## Discussion

The general objectives and structure of a leadership ethics curriculum remain constant, with specifics changing depending on the organization and intended participants. Given the goals of leadership ethics, the curriculum for leaders across our institution consists of 3 to 5 learning modules, each requiring: 0.75 hours of asynchronous review of interdisciplinary, preparatory materials; 1.5 hours of synchronous lecture, discussion, and practice activity; followed by individual debrief sessions of 0.5 hours. Collectively, the modules see participants engage with their core and organizational values, as well as with relevant ethical decision-making frameworks; appreciate the significance of ethical leadership; and practice making ethically defensible decisions using experiential learning methods. The primary obstacle to employing most experiential learning exercises—such as simulations, role plays, and games—is that the time, resources, and expertise required to implement them can be significant.^26^ Accordingly, the leadership ethics curriculum at our institution has participants engage with cases rooted in composite real-world scenarios that our leaders have faced in recent years.

One shortcoming of our curriculum development process was that the needs assessment was not specifically focused on a *leadership ethics* curriculum, but a *leadership* curriculum. In response to this and to further ensure curriculum relevance to participants, the final readings and logistical details of the curriculum are collaboratively finalized by the instructors and participant-cohorts 3 months before the delivery of each course, with the opportunity to add materials in real-time should new issues be raised during the modules. This ensures that each course is tailored to the needs, availability and pedagogical format preferences of each participant group. Sample topics proposed for our senior and director-level leaders have been ransomware, equity regarding human resource issues, priority setting, integration of artificial intelligence, and compensation. Given the variety of possible topics and needs of potential participants, the questions “Who should be the teacher or educator?” and “What should that teacher do so that participants can learn?” is a feature that will be tailored to each setting and cohort, depending on their resources and experiences with psychological safety.

The successfully assisted course participant is one who is better equipped, and more confident, in their ability to engage and make ethically defensible decisions. Evaluations of our leadership ethics course address (1) participant’s experience of the course, (2) extent to which the participant feels the curriculum objectives were met after completing the modules, and (3) extent to which the participant feels the curriculum was useful, after having engaged with a complex ethical issue in their leadership role after the course completion.

## Conclusion

In recent years, leaders in healthcare have faced some of the most ethically challenging issues of their careers. Many health leaders have found themselves in the morally paralyzing situation of being called upon to make values-based decisions for which few have had specific training. Contemporary complex ethical issues facing health leaders are amenable to the structure of an ethics curriculum. Above, we presented how we developed a leadership ethics curriculum, that is tailorable to the unique contexts of different institutions. We applaud the accountability of those who recognize the value of a contextually tailored ethics curriculum for health leaders and look forward to the further entrenchment of ethics in the roles of leadership across healthcare domains.
